# Novel Programmable Shape Memory Polystyrene Film: A Thermally Induced Beam-power Splitter

**DOI:** 10.1038/srep44333

**Published:** 2017-03-09

**Authors:** Peng Li, Yu Han, Wenxin Wang, Yanju Liu, Peng Jin, Jinsong Leng

**Affiliations:** 1 Center for Composite Materials and Structures, Harbin Institute of Technology, No. 2 YiKuang Street, Harbin, 150080, P.R. China; 2 Research Center of Ultra-precision Optoelectronic Instrumentation, Harbin Institute of Technology, No. 2 YiKuang Street, Harbin, 150080, P.R. China; 3 Department of Aerospace Science and Mechanics, Harbin Institute of Technology, No. 92 West DaZhi Street, Harbin, 150001, P.R. China

## Abstract

Micro/nanophotonic structures that are capable of optical wave-front shaping are implemented in optical waveguides and passive optical devices to alter the phase of the light propagating through them. The beam division directions and beam power distribution depend on the design of the micro/nanostructures. The ultimate potential of advanced micro/nanophotonic structures is limited by their structurally rigid, functional singleness and not tunable against external impact. Here, we propose a thermally induced optical beam-power splitter concept based on a shape memory polystyrene film with programmable micropatterns. The smooth film exhibits excellent transparency with a transmittance of 95% in the visible spectrum and optical stability during a continuous heating process up to 90 °C. By patterning double sided shape memory polystyrene film into erasable and switchable micro-groove gratings, the transmission light switches from one designed light divided directions and beam-power distribution to another because of the optical diffraction effect of the shape changing micro gratings during the whole thermal activated recovery process. The experimental and theoretical results demonstrate a proof-of-principle of the beam-power splitter. Our results can be adapted to further extend the applications of micro/nanophotonic devices and implement new features in the nanophotonics.

Combined with thin-film optics, guided-wave optics and diffractive optics, micro/nanophotonic structures have attracted significant attention because of their potential applications as new functional optical components in fields such as optical communication, computing, display technology, anti-counterfeiting efforts and biology. Micro/nanophotonic structures are capable of color control and digital coding, and their diffraction characteristics can alter light to almost any desired intensity profiles or patterns, this capability is known as optical wave-front modification. Their micro/nanostructural units are typically arranged periodically and structurally rigid. Simultaneously, durable and non-deformable materials, such as glass, fused silica, quartz, and acrylates, are used as substrates^1^,^2^,^3^. Micro/nanophotonic structures promote the development of electronics and photonics, but they face significant challenges related to their flexibility, versatility and smart switching mechanism. Alternatively, external stimuli, such as electricity, light, ionic concentration and temperature, can induce structural changes in certain materials and thus modulate their optical performance. Many functional micro/nanophotonic structures have been proved to be constructed using various polymers and been extended to a wide range of applications^4^,^5^,^6^,^7^,^8^. Compared with other functional materials, shape memory polymers (SMPs) are promising candidates to achieve tunable light modulation. The unique perfect properties of the SMPs are their memory and ability to recover their original shape in response to external stimulus^9^,^10^,^11^,^12^. SMPs and their composites have been widely used in biomedical applications^13^, actuators^14^, sensors^15^, textiles^16^, aerospace applications^17^, etc^18^,^19^,^20^,^21^.

Recently, the formation and application of the micro-deformation of SMPs have been more concerted and have become the subject of increasing scientific interest^22^,^23^,^24^. Wang *et al*.^25^ reported the novel ability of acrylate-based SMP to memorize and recover their lithographically fabricated nanoscale surface patterns. Li *et al*.^26^ exploited mechanical instability in SMP membranes consisting of a hexagonal array of micron-sized circular holes and demonstrated colour switching as a result of pattern transformation. Xu *et al*.^27^ prepared various deformable, programmable and shape memorizing micro-optic components using crosslinked poly (ethylene-co-vinyl acetate). Although some substantial researches have been done so far towards studying the micro/nano shape memory effect and their related optical features, long response time, inferior mechanical properties, simple transformation and short of theoretical support greatly limits their application in the tunable optical devices. The polystyrene-based SMPs, which possess excellent mechanical property, high transparency, good toughness, adjustable glass transition temperature, easy processing and driving and fast response characteristics, have remarkable performance compared with other shape memory polymer materials and are fit for optical applications. Meanwhile, these researchers are more concerned about the “static” origin-deformation-recovery states, the fully dynamic transition process has not been systematically studied.

In this study, some dual-side polystyrene-based SMP (PS-SMP) films with programmable surfaces micropatterns were prepared by a combination facile pouring and hot embossing process; all of the films exhibited excellent optical transparency, stability and micro/nanoscale shape memory effects. Using the micro-groove grating patterned on the PS-SMP film, the mechanism of a splitter is discussed by investigating the optical response of the pattern-variations during the whole thermal activated recovery process. The micropatterns of the polymer surface can be easily transformed into any desired display format by predesign. More complicated micropatterns also exhibited the potentially sophisticated beam-power splitter effect.

## Results and Discussion

### Fabrication and characterization of SMP film with micropatterns

**Figure 1:**
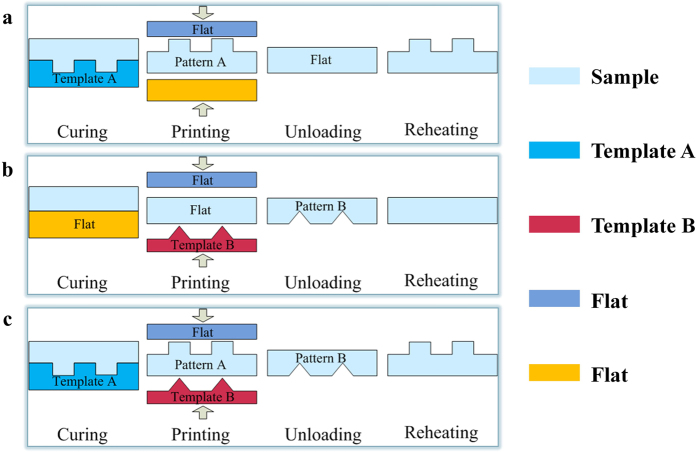
Scheme of the manufacturing and transformation processes for surface micropatterns on an SMP film. (**a**) 1D grating–Flat surface cycle, (**b**) Flat surface–2D grating cycle, (**c**) 1D grating–2D grating cycle.

Micropatterned SMP films were prepared using polystyrene-based SMP (*T*_*g*_ = 67 °C) as the raw material, which are polymerized by styrene with vinyl compound, cross-linking agent (bifunctional monomer), polystyrene (modifier) and benzoyl peroxide (reaction initiator). Compared with the base SMP materials (described by Leng *et al*.^28^ in 2010, *T*_*g*_ = 53.7 °C), the modified polystyrene-based SMP was superior in forming thin films. Through properly adjusting selection of materials’ reasonable ratio and stimulus methods^29^, the polystyrene-based SMP possesses high transparency, good toughness, adjustable glass transition temperature, easy to drive and fast response characteristics. The FTIR spectra of modified styrene-based SMP film (see Fig. S4) and the base SMP materials are identical. The corresponding position and band width of absorption peaks are not drifted, thereby serving as a proof that the chemical groups and bonds characterization had not been changed. A schematic representation of the process of manufacturing and transforming of the micropatterned SMP surfaces is presented in Fig. 1.

Several SMP films with programmable surface micropatterns were prepared using the typical forming methods (pouring forming or hot embossing), which are simple and reliable fabrication approaches that are capable of creating sub-50 nm features at a low cost. Micropatterns that could be easily released from silicon wafers with high precision were transferred onto the SMP surfaces, attributed to the fluidity of the solution and the plasticity of the solid SMP materials.

**Figure 2:**
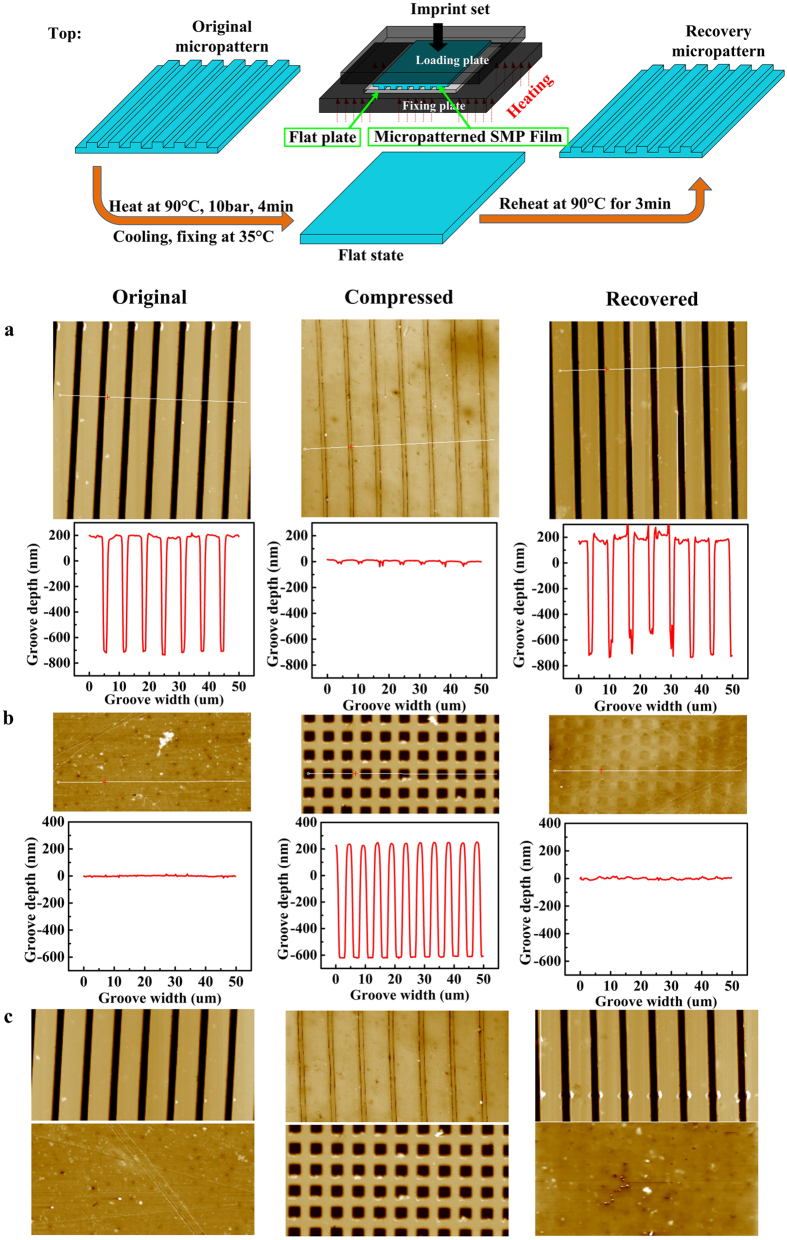
Top: schematic of the deformation and recovery process and the particular imprint set. Shape changing and recovery cycles of the surface micropatterns of the SMP films characterized using AFM.(**a**) 1D grating–flat–1D grating cycle on one side, (**b**) Flat–2D grating–flat cycle on one side, (**c**) 1D grating–2D grating–1D grating cycle on both sides.

Figure 2 shows the three types of films (thickness of 1 mm) and their shape-changing and recovery cycles between a typical groove grating (1D grating) and a chessboard grating (2D grating). The Fig. 2(top) illustrates the deformation and recovery process and the particular imprint set in the Fig. 2a. The process conditions of the Fig. 2b,c are similar, but the micropattens of the loading plate and fixing plate are changed. Firstly, the original 1D grating on the SMP surface, with a pitch of 125 grooves/mm, a groove width of 3.5 μm and a groove height of 900 nm that was formed via the pouring process. The grating-structured SMP film was imprinted using smooth quartz glass at 90 °C and 10 bar for 4 min to cause it to enter a featureless, flat state. The pressure from the glass flattened the SMP surface, which became fixed as the film was cooled to 35 °C before the pressure was released. As Fig. 2a shows, the roughness of the flattened SMP was 15 nm, meaning that the SMP demonstrated excellent memory ability. After the sample was reheated at 90 °C for 3 min, the original grating structure was rapidly recovered. Atomic force microscope (AFM) measurements indicated that, the recovered groove height was approximately 880 nm, representing perfect microstructural recovery. Secondly, the surface of a smooth SMP film that was formed into a temporary 2D grating micropattern with dimensions of 2 μm × 2 μm × 870 nm via the hot embossing method. By reheating the film to temperatures above *T*_*g*_, the original smooth surface could be recovered. Thirdly, Fig. 2c is a combination of Fig. 2a,b that illustrates the transformation of the polymer surfaces on both sides from a 1D grating to a 2D grating. The 1D grating collapsed evenly with a roughness of 30 nm, whereas the 2D grating transformed into a perfectly flat surface.

The experimental results successfully demonstrate the transformations of micropatterns of one, two or multiple dimensions. The two unique sides of the SMP film with switchable micropatterns provide novel features based on the exploitation of the high transparency of the SMP material to demonstrate the desired tunable optical display effects.

The thermal decomposition temperature is up to 300 °C, the TGA curve of the material is given in supplementary materials (Fig. S1). The dynamic structure changed of the SMP grating is controlled under the *Tg* + 20 °C (about 90 °C), which remains far below its decomposition temperature (300 °C). The force-controlled thermo-mechanical properties of shape memory cycles were characterized using a three point bending fixture at force control mode, as shown in Fig. S2. Seven shape memory cycles were performed to examine the repeatability. The sample exhibited good mechanical properties, which the fixity and recovery ratio are all above 95%. Therefore, the material possessing excellent shape memory effect and cyclic fatigue resistance can be used as the dynamic tunable reused optical components.

**Figure 3:**
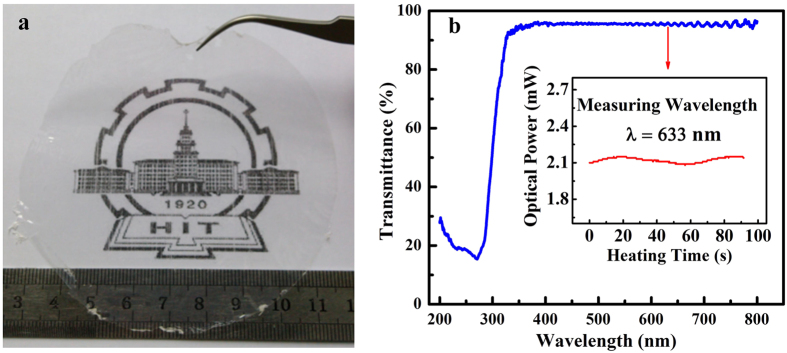
(**a**) Display of “HIT” logo underneath smooth SMP film, (**b**) Ultraviolet and visible light transmittance spectra of a transparent SMP film (The inset shows the change in transmitted optical power from 633 nm light source during the heating process).

For rapid response, thin SMP testing films are preferred for use in optical response experiment. To obtain SMP films with controlled, uniform, ultrathin and dense structures and smooth surfaces, the SMP was dissolved in the solvent and the evaporation was accelerated by a typical spin coating approach. The thicknesses of these SMP films could reach a dozen or even dozens of micro-meters for use in various practical integrated applications. Moreover, the smooth SMP films (thickness of 50 μm) exhibited excellent transparency (Fig. 3) with a transmittance of 95% in the visible spectrum. Thermo-stability studies were also performed by recording the variation in the transmittance of optical power during a continuous heating process up 100 °C using a measuring wavelength of 633 nm. No significant change in optical power was observed throughout the entire heating process.

### Diffraction efficiency measurements of SMP film

**Figure 4:**
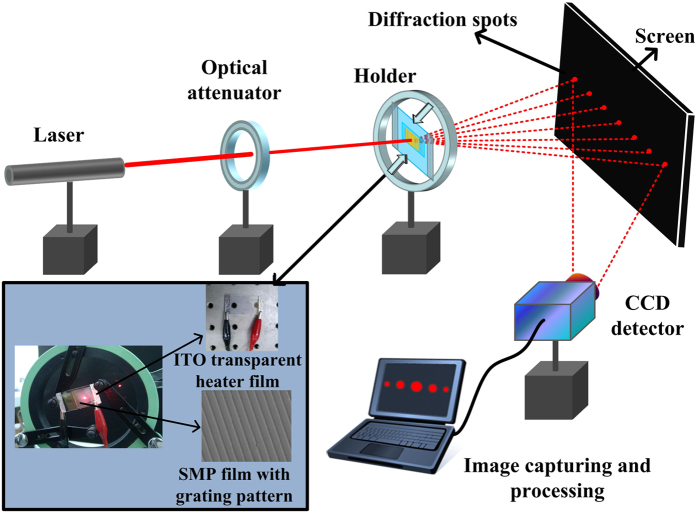
Experimental setup for investigation of the optical response of an SMP film with transmission groove grating structures induced by dynamic thermal actuation.

In subsequent experiments, we exploited the switchability of the SMP film with micro groove grating patterns at the microscale and used diffraction efficiency measurements to demonstrate the material stability, the fabrication precision and the flexibility of the switching between micropatterned and flat surfaces. Figure 4 depicts the experimental setup for the optical response measurements. A visible laser (*λ* = 633 nm) was used as the light source for further investigation of the changes in the optical properties of the SMP film. An optical attenuator was mounted in the light path to regulate the incident light intensity. The SMP film was installed on a transparent ITO electric heating film (5 cm × 3 cm, sheet resistance of approximately 55 Ohm/sq), which was immobilized with an adjustable claw-shaped holder to ensure the perpendicular transmission of the light. The light penetrating through the SMP film and the transmission spots were cast on a black screen set 0.6 m away from the film. A CCD detector that was positioned very close to the film holder and connected to a computer processor was used to detect any changes in the transmission spots.

When a monochromatic light beam encounters a periodic obstruction with an interval comparable to the wavelength of the light, the beam is scattered in various directions, or to various diffraction orders, which can be observed as a regularly patterned distribution. The optical transmission power and diffraction pattern distribution are determined by the characteristics of the micropatterns and their placement on the surface of the SMP film. The diffraction patterns from SMP films with different groove grating patterns were observed. When the SMP films were hot-pressed and made transparent, the transmitted optical patterns were consistent with the original beam shape (laser source). Through adjustments to the optical path, it was ensured that the transmission spot was the only point recorded in the middle of a black screen, and the spot intensity differed little from the incident intensity. By applying a direct voltage (~15 V) to an ITO heating film, we reheated the compressed transparent SMP film (attached on ITO). During the initial stage of heating, the shape and intensity of the transmission spot remained stable. Under a continuously applied voltage, approximately 90 s later, the transmission light spot became hazy and more diffraction spots gradually appeared on the screen. A CCD detector captured the change in the diffraction spots. During the transformation and recovery process of the micropattern on the SMP film surface, the transmission spot produced by the film changed from one spot to various complex diffraction patterns, depending on the design and dimensions of the micropattern.

### Theoretical model

**Figure 5:**
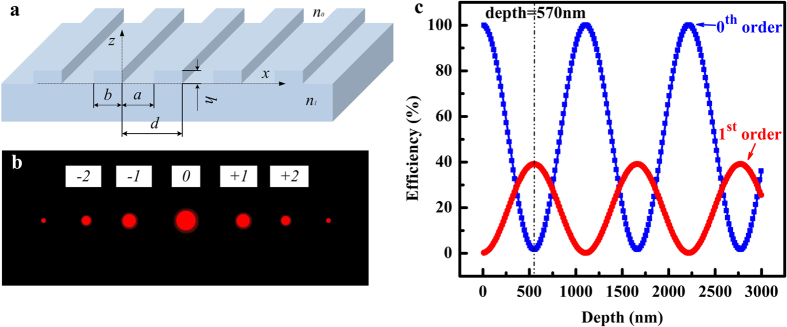
(**a**) Prototype groove grating, (**b**) Its corresponding diffraction pattern, and (**c**) The theoretical relationship between the groove depth and the diffraction efficiencies of 0th and 1st order.

According to the shape-memory mechanism of the SMP, the original micropatterns on the SMP film recover gradually with increasing heating time. In the device design, we introduced into a degree of freedom: the groove depth *h*, which is determined by the etching depth of the silica mold. Figure 5 shows a prototype groove grating, its corresponding diffraction pattern and the theoretical results for its efficiency as a function of the groove depth^30^. For integral values of *m*, the diffraction efficiency from the grating is given by Eq. 1. Here, integer m is the diffraction order (…, −2, −1, 0, 1, 2, …), *d* is the period of the grating, and a is the groove width. The parameter *n*_*0*_ is the index of the environment, *n*_*1*_ is the index of the substrate, and Δ*φ* = 2π*h(n*_*1*_*−n*_*0*_)/*λ* is the phase depth. Specifically, when the device is placed in the atmosphere (*n*_*0*_ = 1), the operating wavelength chosen is *λ* = 632.8 nm, the substrate is PS-SMP (*n*_*1*_ is ~1.57), the feature dimensions are designated as *d* = 8 μm, *a* = 3.5 μm, and the groove depth h (from 0 to 3 μm) is the introduced degree of freedom. When *m* = 0 or 1, the efficiency of the 0th order and 1^st^ order can be calculated according to Eqs 2 and 3.


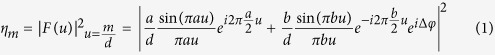



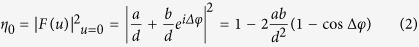



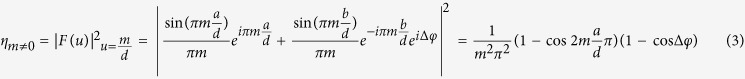


Figure 5c shows the theoretical results for the efficiency as a function of the groove depth. The diffraction efficiency exhibits a periodical change with increasing groove depth, and the 0th order and 1^st^ order efficiencies change inversely in the variation direction. The efficiency of the 0th order declines monotonically from 100% to 0 when the groove depth varies locally within 0–570 nm. The corresponding efficiency of the 1st order occurs at the same time and grows to a steady value of approximately 40%. The proposed model was verified through comparison of the theoretical and experimental results. Moreover, other pattern parameters such as groove width can also be used to design the diffraction efficiency. While we don’t present it, since laterally deforming or dynamic driving thin SMP grating films (tens of microns thick) turns to be a difficult technique to tune width.

### Mechanism

To quantitatively discuss the effects of the optical response of the SMP film on the dynamics of its surface features during the thermally stimulated recovery process, the transformation of the spots was recorded using a CCD detector at a rate of 9 fps, and the corresponding spot energies were calculated using a computer processer. The temperature of the ITO heating film was simultaneously recorded using a thermal infrared imager. An SMP film with a groove grating pattern (125 grooves/mm, groove width of 3.5 μm and *H*_*depth*_ = 600 nm) was prepared and compressed into a flat surface; the temporarily transparent film was then mounted in the light path. The maximum transmission energy of 0^th^ order was measured at the state ‘flattened’, in which no diffraction was observed.

**Figure 6:**
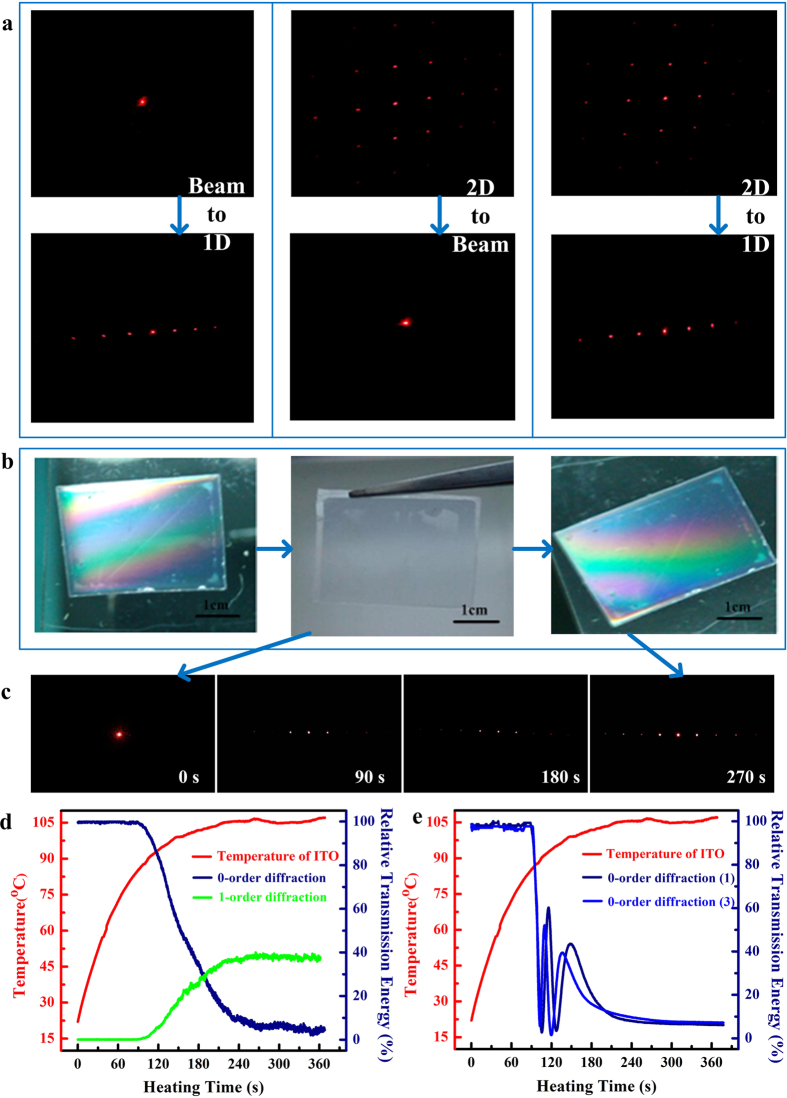
Experimental results of diffraction efficiency measurements. (**a**) The variation in the diffraction pattern distributions of the SMP films during the reheating process (see Visualization 1–3). (**b**) Photographs of the shape memory cycle in the grooved grating structured SMP film under white light illumination (**c**) Real images of the transformation of the diffraction spot during the recovery of the flattened SMP film at different heating times. (**d**) Comparison of the relative transmission energy of 0th-order and 1st-order diffraction spots for the original SMP film with a groove grating pattern (depth of 600 nm). (**e**) 0th-order diffraction of the SMP film (with identical pitch but varying depth of 2.8 μm) repeating three successive shape memory cycles during the ITO heating process.

**Figure 7:**
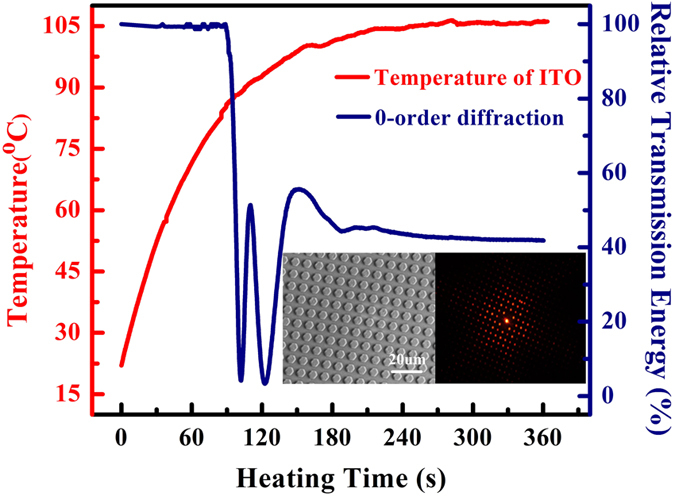
The relative transmission energy of the 0th-order diffraction of an SMP film with hexagonal prism during the heating recovery process (The inset shows the recovered micropatterns and the corresponding diffraction patterns).

For facilitate comparative analysis using theoretical calculations, experimental results were normalized to relative values to the maximum transmission energy, thus a 100% relative transmission energy could be obtained as demonstrated in the Figs 6d,e and 7. Figure 6d shows the changes in the relative power of the 0^th^ and 1^st^ order diffraction spots with heating time and the ITO heating curve. The ITO temperature increased quickly from room temperature and became stable at approximately 100 °C when driven by a 15 V direct voltage.

During the ITO heating process, the intensity of the 0^th^ order diffraction spot changed little as a function of the temperature during the initial stage. As the temperature increased, the intensity suddenly decreased and then eventually stabilized.

Throughout the entire heating process, the relative transmission energy of the 0^th^ order diffraction spot exhibited a steep decline from 100% to ~7%, while the corresponding 1^st^ order diffraction energy exhibited a growth from 0 to ~40%. Referring to the theoretical results above-discussed, the final recovered state of SMP film meet with a grating film with groove height of ~570 nm; and such a diffraction grating shows an efficiency of 40% for 1^st^ order diffraction spot but no 0^th^ order diffraction spot. We can conclude that the deformed groove grating exhibits excellent shape recovery ability (shape recovery ratio (*R*_*r*_) of ~95%) and a dynamic beam-splitter can be achieved with careful design. Furthermore, the ratio between the 0^th^ and 1^st^ order transmission energies can be varied by modifying the groove depth. To study the diffraction effect of the micropatterned SMP films in greater detail, a more deeply groove grating patterned SMP film (*H*_depth_ = 2.8 μm) was prepared and compressed for use in the previously described experiment. Figure 6e shows that the relative power transformation of the 0^th^ order of diffraction exhibited a trend similar to that of the previously mentioned sample in the initial and stable stages. The dynamic response of the optical properties and the heating time exhibited a more complicated trend, which followed a sine wave fluctuation.

The experimental results revealed a clear regularity in the power variation, consistent with the theoretical trend of the 0^th^ order diffraction efficiency cure shown in Fig. 5c. We can see that the dynamic efficiency curve terminated at ~8%, which refers to a recovered groove height of ~2.68 μm and also demonstrates a good shape recovery. However, one thing must be declared here is that the experimental efficiency peak cannot reach at perfect 100% during the recovery process of SMP film, but only ~70% was obtained. We hypothesis that, the dissipated energy by light scattering through ITO increased when it was heating at the high temperature for a long time. Moreover, the switching between transmission patterns was successfully repeated over several cycles. Cross-validation and replication of the experiments revealed that the SMP films exhibited excellent specificity and stability. The plot of the 3^rd^ cycle (blue curve) presented in Fig. 6e shows that the SMP exhibited a good repetitive shape memory effect. Because of the Gaussian distribution of the laser beam used in holographic lithography and the possibility of minor misalignment of the optics, different nanostructure distributions may exhibit slight variations in their influence on the 0^th^ order diffraction optical power attenuation and pattern distribution. Therefore, the micropatterns to be distributed on an SMP film must be properly selected for practical attenuation, splitting and switching needs.

During the deformation of the groove grating (when the micropatterns disappeared) and its recovery (when the micropatterns reappeared), the SMP film was observed to reversibly switch from a transparent state to a colored state upon being illuminated with white light (Fig. 6b). This phenomenon demonstrated the shape memory effect of the micropatterns induced by the optical field. SMP films with different micropatterns all exhibited similar effects because of the shape memory of micropatterns and the optical diffraction effect. In the propagation direction, the transmission properties also changed during the deformation and recovery cycle. Figure 6a shows the change in the diffraction pattern that occurred upon illumination with monochromatic light, corresponding to the procedure represented in Fig. 1a–c. Supporting movie S1,S2,S3 show three switchable diffraction patterns that were exhibited by the SMP films during the reheating process. To evaluate the switchable effects of more complex micropatterned SMP surfaces, we also investigated the relative transmission energy of the 0^th^ order of diffraction of an SMP film with a hexagonal prism pattern (array period of 30 μm × 30 μm, inner radius of 10 μm, and height of 600 nm) during the heating revoery process as confirmation of the switching mechanism (Fig. 7).

When the experiment described above was repeated with a film with a micro-hexagonal prism pattern, this film also exhibited an excellent shape-memory effect and diffraction effect. The beam energy remained at a stable high value of approximately 40% throughout the damping transition. These results provide excellent evidence supporting the feasibility of customizable design for dynamic beam power splitting applications.

## Conclusions

In conclusion, facile lithographic replication and suitable materials offer exciting opportunities for the fabrication of switchable beam splitter to promote the development of adaptive photonic devices. We have shown that thermally-activated PS-SMP beam-power splitter films with switchable optical properties can be obtained by typical pouring forming and hot embossing processing. By patterning micro-groove gratings on the SMP films, three types of the optical beam film splitter were designed, fabricated and investigated based on the mechanical properties, shape memory and optical diffraction effect. For insight into how light can be effectively manipulated through SMP film, the diffraction efficiency was qualitatively and quantitatively analysed throughout the entire thermal recovery process. Significantly, we envision the presented optical transformation properties and matrix materials to have a wide impact, not exclusively in thermal actuation and one-way switching, but also provide a wide range of opportunities for the combination of suitable manipulate materials functionalities, including the various actuation (optic, electric, magnetic, acoustic *et al*.), two-state transformation even multi-state conversion in adaptive optical systems with more complex functions.

## Methods

### Materials and templates

Micropatterned SMP films were prepared using polystyrene-based SMP (*T*_*g*_ = 67 °C) as the raw material, which was produced in Prof. Leng’s laboratory. Silicon wafer surfaces with micro/nanostructures were customized by the Research Center of Ultra-precision Optoelectronic Instrumentation.

### Sample preparation

The fabrication of the controlled uniform thin SMP film with programmed micriostructures was performed in four consecutive steps: (i) a silicon wafer template with designed microstructures, which was washed with acetone, ethyl alcohol and water before use, was prepared and mounted on the tray of the spin coater. A polystyrene-based SMP solution (1 mL) was dispensed onto the silicon template, which was tilted and rotated to spread the solution over the entire wafer. The wafer was spin-coated at 500 rpm/s for 9 s, and then quickly accelerated to 1000 rpm/s for 20 s to obtain a uniform thickness, a dense structure and a smooth surface. The microstructure was transferred onto an SMP film through the spin coating process. The thickness of the film was dependent on the viscosity of the PS-SMP solution and the spin speed used for coating. (ii) After spin-coating, for wet-forming, the wafer was transferred to a glass container of ethanol for 30 s to accelerate the transition between the crystal morphology and stripping. (iii) The wafer was subsequently removed and stored in an empty glass container for 2 h at 75 °C. The films were cured after being pre-baked to cause the solvent to evaporate. (iv) The cured resin formed a clear film after a lift-off process. The microstructured SMP film was carefully peeled off after curing, resulting in a complementary topographic surface structure on the original wafer.

To prepare ordinary photonic polymer samples, the pre-polymer solution was spread over the template, and after curing at 75 °C for 24 h, the resulting cross-linked polymer with replicated structures was peeled off from the nanostructured slide.

Transparent ITO heater films with dimensions of 50 mm × 30 mm × 1 mm were obtained from Sigma-Aldrich. Al electrodes were bonded at each end of the ITO film to allow for voltage application. Considering the heating efficiency and experimental response, we selected 15 V as the heating voltage.

### Optical and structural characterization

The microstructured SMP surfaces were observed using field emission environmental scanning electron microscopy (SEM, Quanta 200 FEG), AFM (Dimension Icon) and optical microscopy (VHX-900). Fourier transform infrared spectroscopy was performed to identify the changes in functional groups of the SMP film. All the samples were scanned from 4000 to 400 cm^−1^ using a medium scan speed. The transmission spectra of the SMP films were obtained using a UV-VIS-NIR spectrophotometer (Shimadzu UV-2550). The optical properties of the SMP film were characterized based on their transmittance spectra. A visible laser (λ = 633 nm) was used as the light source for the diffraction measurements. The light transmitted through the SMP film was focused on a screen, and the optical power was recorded by a photo detector (Thorlabs) and a CCD camera.

### Thermal Gravimetric Analysis (TGA)

To illustrate the thermal property, the thermal gravimetric analysis (TGA) was carried out from 25 °C to 600 °C using TGA/DSC 1 STARe System (Mettler-Toledo AG Analytical, Switzerland).

### Shape Memory Behavior

Shape memory ability of SMP film was measured in height of the micropatterns using AFM. An original micropattern on the SMP film surface with an initial height *H*_*0*_ was first compressed at 90 °C. The compressed film was then removed from hot compressing machine and allowed to achieve an equilibrium height of *H*_*1*_. The recovery efficiency of the SMP was measured by reheating the unloaded film in its temporary shape for 90 °C and allowing the sample to recover to height *H*_*2*_. The shape recovery ratio *R*_*r*_ was defined by Eq. 4.


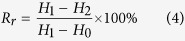


To further illustrate the shape memory cycle times, the force-controlled thermo-mechanical properties of shape memory cycles were characterized on a Dynamic Mechanical Analysis (DMA) Q800/RSA3 (TA Instruments, America) using a three point bending fixture at force control mode. The rectangular sample was first heated to 90 °C and kept in an isothermal condition for 5 min. The sample was then three point bended at 90 °C from its “permanent” shape at the beginning of the Nth testing cycle to the bended shape under a final pressure of 0.25 N. It was then cooled to 35 °C with the force kept constant. After being held at 35 °C for 5 min, the applied force was released to the preload force 1 mN. Finally, the temperature was ramped from 35 to 90 °C and kept isothermal for 5 min. Seven shape memory cycles were performed to examine the repeatability.

## Additional Information

**How to cite this article**: Li, P. *et al*. Novel Programmable Shape Memory Polystyrene Film: A Thermally Induced Beam-power Splitter. *Sci. Rep.* **7**, 44333; doi: 10.1038/srep44333 (2017).

**Publisher's note:** Springer Nature remains neutral with regard to jurisdictional claims in published maps and institutional affiliations.

## Supplementary Material

Supporting Movie S1

Supporting Movie S2

Supporting Movie S3

Supplementary Information
