# Supplementary material for: Novel Programmable Shape Memory Polystyrene Film: A Thermally Induced Beam-power Splitter
Source: Sci Rep. 2017 Mar 9;7:44333. doi: 10.1038/srep44333 (PMC5343575; doi:10.1038/srep44333)
Supplement: Supplementary Information [file srep44333-s4.pdf]

Novel Programmable Shape Memory Polystyrene Film: A Thermally Induced Beam-power Splitter (Supplementary Information)

Peng Li[1], Yu Han[2], Wenxin Wang[1], Yanju Liu[3], Peng Jin[2,*], and Jinsong Leng[1,*]

[1]Centre for Composite Materials and Structures, Harbin Institute of Technology (HIT), No. 2 YiKuang Street, Harbin, 150080, P.R. China.

[2]Research Center of Ultra-precision Optoelectronic Instrumentation, Harbin Institute of Technology (HIT), No. 2 YiKuang Street, Harbin, 150080, P.R. China.

[3]Department of Aerospace Science and Mechanics, Harbin Institute of Technology (HIT), No. 92 West DaZhi Street, Harbin, 150001, P.R. China.

Correspondence and requests for materials should be addressed to Jinsong Leng (lengjs@hit.edu.cn) or Peng Jin (P.Jin@hit.edu.cn)

Here, for additional clarification, we provide more detailed discussions and further data for which there was no space in the main text of our manuscript.

Thermal Gravimetric Analysis (TGA)

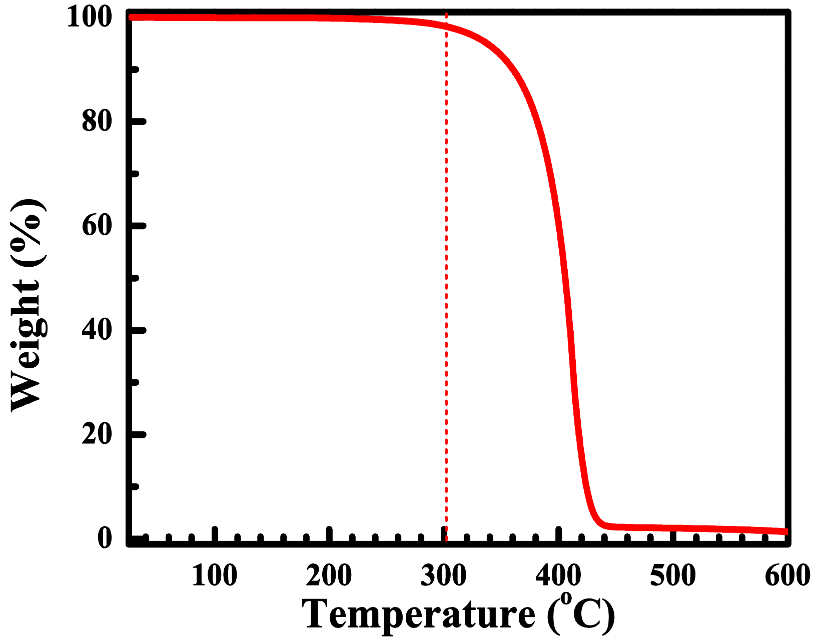

Figure S1. The thermal gravimetric analysis (TGA) curve of the polystyrene based shape memory polymer sample.

Dynamic Mechanical Analysis

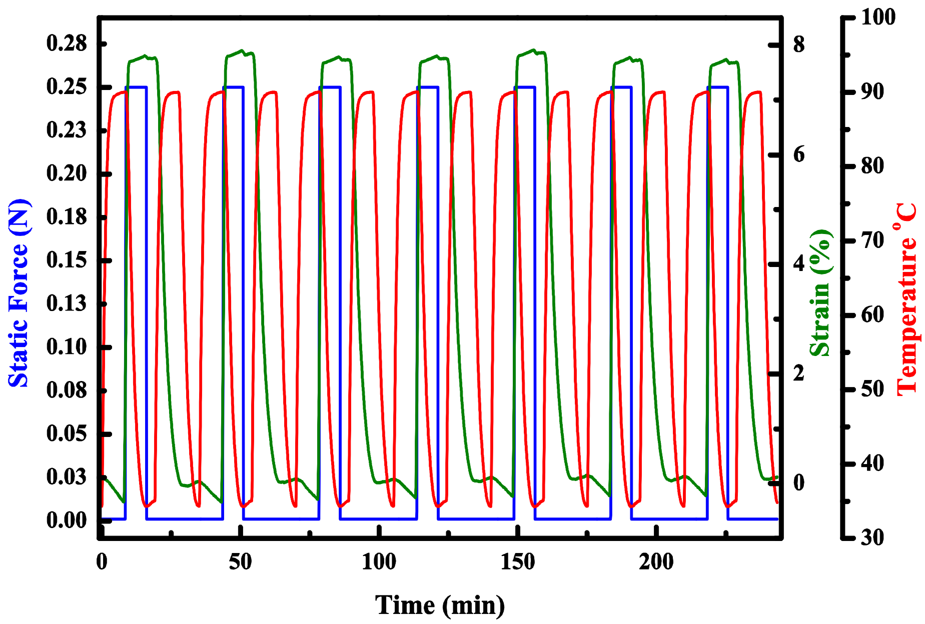

Figure S2. Stress-controlled cyclic thermo-mechanical testing curves of the polystyrene based shape memory polymer sample.

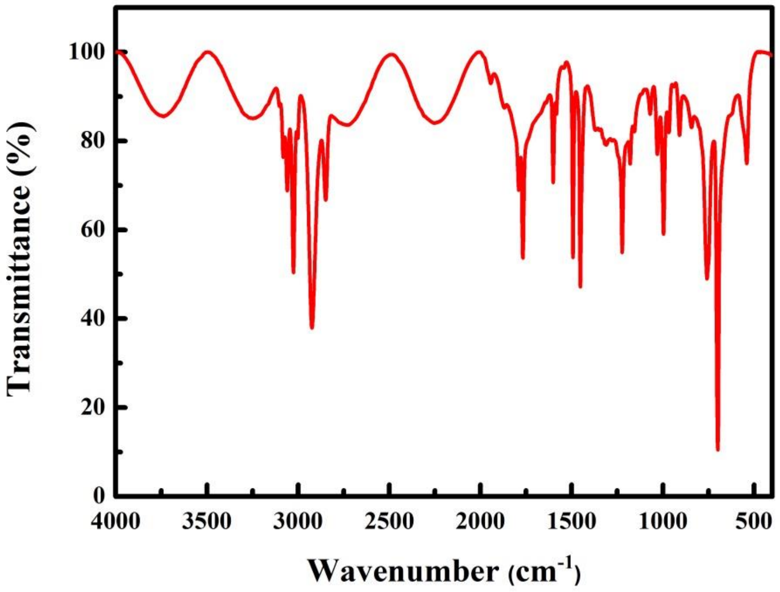

Figure S3. FTIR spectra of styrene-based SMP film

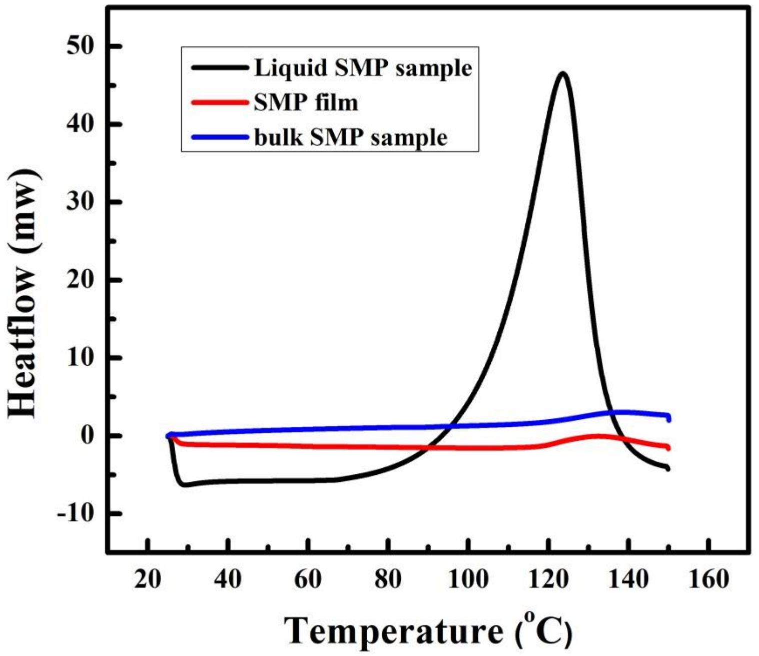

Figure S4. Engineering stress-strain curves of SMP samples

Supplementary Movies:

Supporting Movie S1:

The switchable variation in the diffraction pattern distributions of the SMP samples during the reheating process from the original beam to a different single direction.

Supporting Movie S2:

The switchable variation in the diffraction pattern distributions of the SMP samples during the reheating process from two directions to the original beam.

Supporting Movie S3:

The switchable variation in the diffraction pattern distributions of the SMP samples during the reheating process from two directions to one direction.
